# Genetic architecture of obesity and advances in precision pharmacotherapy: a comprehensive review

**DOI:** 10.3389/abp.2026.15484

**Published:** 2026-04-07

**Authors:** Floren Kavaja, Thomas Liehr, Gazmend Temaj

**Affiliations:** 1 Medical Faculty, University of Prishtina, Prishtina, Albania; 2 Jena University Hospital, Friedrich Schiller University, Institute of Human Genetics, Jena, Germany; 3 Human Genetics, College UBT, Faculty of Pharmacy, Prishtina, Albania

**Keywords:** GLP-1 receptor agonists, leptin-melanocortin pathway, monogenic obesity, obesity genetics, polygenic obesity

## Abstract

Obesity, a global health catastrophe, arises from complex interactions between environmental factors and genetic predispositions. This review summarizes the current state of knowledge on the genetic basis of obesity and contrasts rare monogenic forms caused by mutations in a single gene with common polygenic forms caused by hundreds of genetic variants with small effects. We highlight important genes in neuroendocrine signaling pathways, particularly the leptin-melanocortin system involving *MC4R*, *LEP*, and *POMC*, as well as newly identified loci from genome-wide association studies such as *FTO* and *SEC16B*. The interplay between genetic probability and environmental factors underscores the heterogeneity of obesity phenotypes. Recent advances in pharmacotherapy, such as GLP-1 receptor agonists and dual/triple incretin agonists, demonstrate strong efficacy across various genetic backgrounds and underscore the translational relevance of genetic insights. New findings from different groups support the use of polygenic risk scores to identify individuals at risk and suggest prevention strategies. This review discusses the genomic data on clinical practice and emphasizes the possibilities and challenges of precision medicine in obesity treatment. Future research should focus on length of genetic screening and elucidating gene-environment interactions to optimize treatment outcomes.

## Introduction

Obesity as a clinical condition is becoming a growing problem according to data from the World Health Organization (WHO). According to the WHO, around 2.5 billion adults were overweight in 2022, and 890 million of these were obese).^1^ The main factor contributing to obesity is the consumption of high-calorie foods, but other factors also play a role and sometimes even have a decisive influence, such as endocrine dysfunction, genetics, and sleep (Keith et al., 2006). Obesity is associated with multiple comorbidities, including various diseases such as cardiovascular disease (Singh et al., 2013), type 2 diabetes (Singh et al., 2013), non-alcoholic fatty liver disease (Li et al., 2016), and high blood pressure (Wang and Wang, 2004). It has been reported that obesity can double the risk of high blood pressure and triple the risk of type 2 diabetes (DMT2) (Thompson et al., 1999). Environmental factors are certainly involved in the rapid increase in prevalence, which results from the interaction of environmental factors and innate biological factors. There are significant genetic factors involved in body weight variation that determine the response to obesogenic environmental factors. For example, twin studies have estimated the heritability of obesity to be between 40% and 70% (Elks et al., 2012; Loos and Yeo, 2022; Lister et al., 2023).

Following established traditional protocols, Loos and Yeo (2022) divided obesity into two categories. First, there is monogenic obesity, which is based on Mendelian rules associated with a severe disease course and states that underlying mechanisms of obesity would include deletions in the chromosome or the deletion of a single gene Second, there is polygenic obesity, also known as common obesity, which is associated with hundreds of polymorphisms (Loos and Yeo, 2022).

Polygenic obesity, the most common form of obesity, is caused by the additive effects of numerous common genetic variants—current studies identify over 500 loci that contribute to a small extent to interindividual differences in body mass index and fat mass (Loos, 2025; Smit et al., 2025). In contrast to rare monogenic obesity, polygenic obesity involves the cumulative influence of alleles with low penetrance in genes involved in appetite regulation (such as *FTO* and *MC4R*), energy homeostasis, and adipogenesis (Van Uhm et al., 2025). Genome-wide association studies (GWAS) have shown that these variants interact strongly with environmental influences such as diet, physical activity, and socioeconomic status to influence obesity risk (Loos, 2025; Van Uhm et al., 2025). Polygenic risk scores validated in a recent cohort enable the quantification of individual genetic susceptibility and the prediction of obesity in populations studies (Masip et al., 2025; Menon et al., 2024; Saeed et al., 2025). These scores underscore the need to combine genetic stratification with personalized lifestyle. Healthy environmental changes can themselves reduce the genetic risk for obesity, while unfavorable conditions can exacerbate this risk (Van Uhm et al., 2025).

Consanguinity, defined as the connection between individuals who are genetically closely related (Temaj et al., 2022), increases the likelihood of homozygosity for rare pathogenic variants and thus the risk of monogenic forms of obesity, such as those caused by mutations in the *LEP* (leptin) or *LEPR* (leptin receptor) genes. This genetic factor is particularly significant in populations with a high rate of consanguineous marriages, where the probability of mutation of recessive features increases significantly. Population studies have indicated that mutations in genes such as *LEP*, *LEPR*, and *MC4R* may explain up to 30% of cases of severe early-onset obesity. Mutations in *LEP* and *LEPR* follow an autosomal recessive inheritance pattern, leading to extreme, rapid-onset obesity and hyperphagia; these cases are often associated with metabolic and hormonal disorders. The finding of new mutations in the *LEPR* gene in consanguineous families underlines the value of genetic screening in this context, where the clinical feature typically corresponds to leptin deficiency; severe early-onset obesity, intense hyperphagia, and hypogonadal hypogonadism is also possible in these cases (Chaves et al., 2022; Dayal et al., 2018; Niazi et al., 2018).

Genetic research studies on monogenic and polygenic obesity have shown that the underlying biology is based on common neuroendocrine signaling pathways that primarily affect the central regulation of appetite and energy balance of the brain. Loos and Yeo (2022) point out that genes first associated with rare, severe, early-onset monogenic obesity—such as *LEP*, *LEPR*, *POMC*, and *MC4R*—repeatedly share variants that influence body mass index in the general population, suggesting a key role for the hypothalamic leptin-melanocortin signaling pathway in both forms of obesity (Loos, 2025; Loos and Yeo, 2022). Similarly, Trang and Grant (2023) note that genes originally discovered in monogenic contexts, including *MC4R* and *POMC*, overlap with loci identified in large-scale polygenic studies and act via common biological signaling pathways such as BDNF–TrkB (Brain-Derived Neurotrophic Factor–Tropomyosin Receptor Kinase B) and leptin–melanocortin (Trang and Grant, 2023). Hinney et al. (2010) further emphasize that while rare mutations in monogenic obesity cause dramatic phenotypes, the same biological pathways are modulated more subtly by numerous common variants in polygenic obesity, supporting the notion that both genetic architectures ultimately converge on largely similar neural mechanisms for regulating energy homeostasis (Hinney et al., 2010). In this review, we summarize genetic studies that have characterized molecules and mechanisms as well as therapies for controlling body weight.

## The leptin-melanocortin signaling pathway: central mechanisms regulating appetite and energy homeostasis in obesity

**FIGURE 1:**
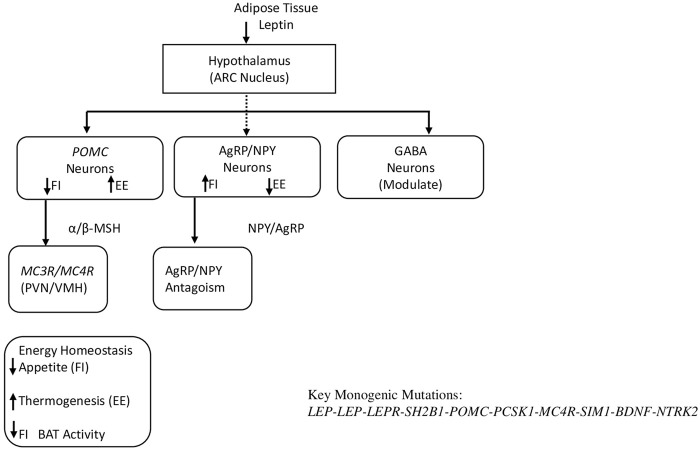
This schematic illustrates the central leptin-melanocortin pathway regulating appetite and energy homeostasis, with monogenic obesity genes positioned along the signaling cascade.

The leptin-melanocortin signaling pathway plays a crucial role in biochemical signaling pathway-related obesity by regulating appetite and energy homeostasis. It is known that leptin is secreted by adipose tissue, binds to leptin receptors (LEPR) in the hypothalamus, and activates intracellular signaling cascades, such as the JAK-STAT and PI3K signaling pathways, to regulate neuronal activity. This process stimulates pro-opiomelanocortin (POMC) neurons to produce α- and β-melanocyte-stimulating hormones (MSH), which subsequently activate melanocortin-4 receptors (MC4R) to produce satiety signals that suppress appetite and increase energy expenditure. At the same time, leptin can inhibit agouti-related peptide (AgRP) neurons and counteract MC4R signaling, thus reducing appetite. Disruptions in any of the components of this signaling pathway, such as mutations in *LEP*, *LEPR*, *POMC*, or *MC4R*, often lead to severe early-onset obesity because they impair these regulatory mechanisms. The role of this pathway in energy balance is confirmed by clinical studies showing altered protein levels of pathway components in obese patients, as well as by experimental models in which loss of function leads to increased food intake and weight gain (Collet and Schwitzgebel, 2024; Farooqi, 2023; Tas et al., 2022; Yeo et al., 2021). Maintaining the integrity of this signaling pathway is crucial for controlling body weight and represents an important target for obesity therapy (Figure 1).

## Gene discovery in obesity

### Gene discovery in monogenic obesity

**FIGURE 2:**
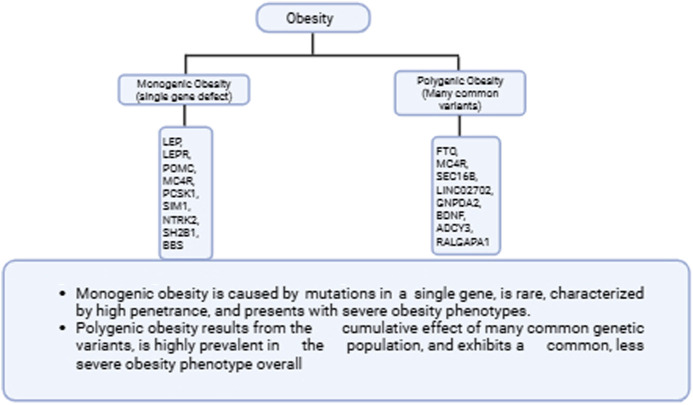
This figure clarifies the main categorical distinction.

The discovery of genes for monogenic obesity began with the identification of rare, highly penetrant mutations that lead to severe early-onset obesity, particularly in genes of the leptin-melanocortin signaling pathway such as *LEP*, *LEPR*, *POMC*, and *MC4R* (Loos, 2025). Targeted panels for the 25–35 genes listed here (including *LEP, LEPR, MC4R, POMC, PCSK1, SIM1,* and *SH2B1*) using NGS show a diagnostic yield of approximately 5%–7% in severe early-onset cohorts (Künzel et al., 2025). Based on the guidelines from ACMG/AMP (Richards et al., 2015), variants should be classified into five categories: Pathogenic (P), Likely Pathogenic (LP), Variant of Uncertain Significance (VUS), Likely Benign (LB), or Benign (B) (Richards et al., 2015). These genes were initially characterized in affected humans and corresponding animal models, demonstrating their central role in appetite regulation and energy homeostasis (Mohammed et al., 2023; Saeed et al., 2023). The leptin-melanocortin pathway is fundamental for maintaining energy homeostasis, as it governs both appetite regulation and overall energy balance within the body. The most commonly affected gene is *MC4R* (melanocortin 4 receptor), whose mutations are the main cause of non-syndromic severe early-onset obesity and account for up to 6% of cases of severe obesity (Künzel et al., 2025). Other important genes are *LEP* (leptin) and *LEPR* (leptin receptor) (Chung, 2012; Nunziata et al., 2019). The genes listed below *POMC* (pro-opiomelanocortin) (Huvenne et al., 2016) are *PCSK1*, *SIM1*, *NTRK2*, and those encoding proteins associated with Bardet-Biedl syndrome (BBS) (Huvenne et al., 2016; Künzel et al., 2025). The rare deletion in chromosomes, such as the 16p11.2 microdeletion, which includes the *SH2B1* gene, are also associated with obesity (Künzel et al., 2025). Taking all of this information together, it is clear these genes underscore the critical role of neuroendocrine circuits in controlling body weight, with monogenic obesity representing severe phenotypes caused by defects in a single gene (Figure 2) (Huvenne et al., 2016; Künzel et al., 2025). Table 1 lists the rare, highly penetrant mutations in important neuroendocrine circuits that primarily affect the leptin-melanocortin signaling pathway and cause severe phenotypes of monogenic obesity.

**TABLE 1:** Main genes responsible for rare (monogenic) obesity.

Gene	Full name	Pathway/function	Typical phenotype	References
*LEP*	Leptin	Leptin-melanocortin; appetite regulation	Severe early-onset obesity, hyperphagia, endocrine effects	Mohammed et al. (2023)
*LEPR*	Leptin receptor	Leptin-melanocortin; appetite regulation	Severe early-onset obesity, metabolic disturbances	Nunziata et al. (2019), Chaves et al. (2022), Mohammed et al. (2023)
*POMC*	Pro-opiomelanocortin	Leptin-melanocortin; precursor hormone	Severe early-onset obesity, adrenal insufficiency	Krude et al. (1998), Mendiratta et al. (2011)
*MC4R*	Melanocortin 4 receptor	Leptin-melanocortin; appetite regulation	Most common, severe non-syndromic early-onset obesity	Sridhar and Gumpeny (2024)
*PCSK1*	Proprotein convertase subtilisin/Kexin type 1	Hormone processing	Severe early-onset obesity, hormonal deficiencies	Ramos-Molina et al. (2016), Stijnen et al. (2016)
*SIM1*	Single-minded family bHLH transcription factor 1	Hypothalamic development	Severe obesity, sometimes associated with neurobehavioral disorders	Jr (2000), Mohammed et al. (2023)
*NTRK2*	Neurotrophic receptor tyrosine kinase 2	BDNF signaling; appetite regulation	Severe obesity, neurodevelopmental symptoms	Gray et al. (2007), Alhamas et al. (2022)
*SH2B1* ^a^	SH2B adaptor protein 1	Leptin/melanocortin & insulin signaling	Obesity with insulin resistance (often due to 16p11.2 deletion)	Perrone et al. (2010), Hanssen et al. (2023)
*BBS* genes^b^	Bardet-biedl syndrome gene family	Ciliopathy; various cellular functions	Syndromic obesity, cognitive impairment, retinal dystrophy	Guo and Rahmouni (2011)

^a^ Rare chromosomal deletions such as 16p11.2 (containing SH2B1) are also implicated in some cases.

^b^ BBS genes represent multiple Bardet-Biedl syndrome genes (e.g., BBS1, BBS2, etc.), which contribute to syndromic obesity.

This table reflects the rare, highly penetrant mutations in key neuroendocrine circuits predominantly involving the leptin-melanocortin pathway, causing severe phenotypes of monogenic obesity.

#### LEP gene

Mutations in the *LEP* gene, which is responsible for encoding the hormone leptin, are a rare but well-documented cause of severe early-onset obesity in humans. Patients with congenital leptin deficiency due to homozygous or heterozygous *LEP* mutations display rapid and uncontrolled weight gain in early childhood, often followed by serious hyperphagia and significant metabolic disorders. Many clinical studies have reported that such mutations impair leptin production or function, disrupting hypothalamic regulation of appetite and satiety and leading to profound changes in energy balance and obesity. Patients with *LEP* gene mutations typically have low or undetectable levels of leptin in serum and may develop complications such as metabolic syndrome, fatty liver disease, or hypogonadism (Strobel et al., 1998; Fischer-Posovszky et al., 2010; Yupanqui-Lozno et al., 2019). It is notable that leptin replacement therapy in these patients can effectively reduce food intake, normalize body weight, and correct associated metabolic disorders, underscoring the central role of the *LEP* gene in human energy homeostasis (Dayal et al., 2018; Zhao et al., 2014).

#### *LEPR* gene

The mutations in the *LEPR* gene (which encode the leptin receptor) are known to cause monogenic severe early-onset obesity. Patients with biallelic pathogenic *LEPR* gene mutations typically manifest marked hyperphagia and fast weight gain beginning in infancy or early childhood. These types of mutation have the ability to disrupt the binding or signal transduction function of the leptin receptor and cause leptin resistance; although the presence of leptin is higher in the blood, the appetite-regulating pathways in the hypothalamus do not respond to the satiety signal, resulting in uncontrolled food intake and severe obesity. Other clinical features which are shown to be commonly associated with *LEPR* deficiency include hypogonadotropic hypogonadism and, to varying degrees, changes in pubertal development or hormonal disorders, while metabolic pathway complications such as type 2 diabetes may also occur (Kleinendorst et al., 2020). Recent studies reported that MC4R agonists such as setmelanotide can significantly decrease weight and hunger in patients with confirmed *LEPR* mutations, underscoring the importance of accurate genetic diagnosis for personalized treatment strategies (Chaves et al., 2022; Nunziata et al., 2019; Supti et al., 2024).

#### *POMC* gene

The gene *POMC* is known to encode pro-opiomelanocortin, and mutations of the *POMC* gene are shown to cause rare but severe forms of monogenic obesity (Künzel et al., 2025). This type of obesity is characterized by rapid, early-onset weight gain, intense hyperphagia, and pronounced endocrine disorders. Patients with homozygous or heterozygous *POMC* mutations usually develop severe obesity within the first year of life, as well as secondary adrenal insufficiency due to ACTH (adrenocorticotropic hormone) deficiency and distinctive traits such as pale skin and red hair arise from inadequate levels of melanocyte-stimulating hormones, resulting in reduced melanin production in the hair and skin. These findings underscore the critical role of *POMC*-derived peptides in regulating many processes in the body such as appetite, energy homeostasis, adrenal function, and pigmentation via the leptin-melanocortin signaling pathway (Yeo et al., 2021). Comprehensive reviews and literature searches often highlight the importance of considering POMC deficiency in cases of severe, early-onset obesity combined with adrenal insufficiency even when characteristic changes in pigmentation are absent, as phenotypic expression can vary widely in individuals with similar genetic mutations (Çetinkaya et al., 2018; Dubern et al., 2008; Mendiratta et al., 2011).

#### *MC4R* gene

Gene mutations in *MC4R* are a common form of monogenic obesity (Giannopoulou et al., 2026) and are characterized by early-onset severe obesity, hyperphagia (increased appetite), and metabolic disorders such as hyperinsulinemia and dyslipidemia. The gene *MC4R* encodes the melanocortin-4 receptor, a G-protein-coupled receptor that plays a pivotal role in many signaling pathways such as hypothalamic leptin-melanocortin, which is responsible for regulation of energy balance by suppressing food intake and increasing energy expenditure (Imangaliyeva et al., 2025). Both heterozygous and homozygous mutations in the *MC4R* gene have been documented; patients with homozygous mutations frequently experience a significantly more severe clinical presentation compared to those with heterozygous variants. Individuals with *MC4R* mutations often show rapid weight gain, impaired satiety, and increased linear growth in infancy or early childhood. The variable penetrance and expressivity of *MC4R* gene mutations have shown to influence clinical severity, but the primary features remain consistent. Targeted therapies such as the *MC4R* gene agonist setmelanotide have been reported to offer clinical benefit by reducing hyperphagia and promoting weight loss in affected individuals. Despite great advances, treatment remains multifaceted and includes lifestyle interventions and new pharmacotherapies tailored to genetic diagnosis (Doulla et al., 2014; Imangaliyeva et al., 2025; Sridhar and Gumpeny, 2024; Wade et al., 2021).

#### *PCSK1* gene

The *PCSK1* gene is responsible for producing the enzyme proprotein convertase 1/3 (PC1/3). Mutations in the *PCSK1* gene are responsible for rare but severe forms of monogenic early-onset obesity (Verde et al., 2025). This type of enzyme is crucial for the proteolytic processing of various neuropeptides and prohormones, including those involved in the regulation of appetite, energy balance, and glucose metabolism, such as proopiomelanocortin and proinsulin. The PCSK1 p.Asn221Asp (c.661 A > G) variants have been associated with metabolic pathways, fatty acid oxidation, and hormone processing, increasing the risk of obesity (Verde et al., 2025). Loss-of-function mutations in the *PCSK1* gene result in impaired cleavage of these hormone precursors, leading to hyperphagia, severe obesity, and multiple discordance of the endocrinopathies, such as adrenal insufficiency, hypogonadism, and malabsorptive diarrhea in infancy. Both homozygous and heterozygous mutations can cause a spectrum of clinical features, ranging from extreme obesity with neuroendocrine dysfunction to milder phenotypes in heterozygous carriers. These findings underscore the crucial role of PCSK1 in energy homeostasis and endocrine regulation in humans (Löffler et al., 2017; Ramos-Molina et al., 2016; Stijnen et al., 2016).

#### *SIM1* gene

*SIM1* gene mutations are responsible for rare forms of monogenic obesity (Mohammed et al., 2026), characterized by early-onset severe obesity, hyperphagia, and, frequently, neurological behavioral abnormalities such as developmental delays, intellectual disability, and autism. The *SIM1* gene is responsible for encoding a transcription factor which is essential for neuronal development in the paraventricular nucleus of the hypothalamus, a brain region critical for the regulation of appetite and energy balance (Mohammed et al., 2026). Loss-of-function mutations reduce *SIM1* transcription activity, leading to hypothalamic dysfunction, increased food intake, and, thus, severe obesity. Some affected individuals show features of Prader-Willi syndrome, such as hypotonia and facial dysmorphia, although this phenotype is variable. Many family studies shave shown that rare *SIM1* gene variants contribute significantly to familial obesity risk, with mutation severity and environmental factors influencing phenotypic expression. These findings confirm the central role of *SIM1* in neurodevelopment and energy homeostasis and mark it as a key gene for genetic screening in early-onset obesity (Bonnefond et al., 2013; Ramachandrappa et al., 2013; Stanikova et al., 2017).

#### *NTRK2* gene

The gene *NTRK2* is responsible for the encoding of tropomyosin receptor kinase B (TrkB) (Roussel-Gervais et al., 2023), and mutations in this gene have been associated with severe early-onset obesity characterized by hyperphagia and a disturbed energy balance. The receptor kinase TrkB plays a pivotal role in the brain-derived neurotrophic factor (BDNF) signaling pathway, particularly in hypothalamic neurons that regulate appetite and energy expenditure (Roussel-Gervais et al., 2023). Loss-of-function mutations or deletions of the *NTRK2* gene in key hypothalamic regions such as the paraventricular nucleus increase food intake, decrease physical activity, and cause rapid weight gain. Functional studies of human *NTRK2* mutations, such as the Y722C variant, show impaired receptor signaling and neuronal function, which may contribute to deficits in hypothalamic neurogenesis and pronounced obesity phenotypes. Furthermore, selective deletion of *Ntrk2* in hypothalamic neurons of mice reproduces hyperphagic obesity, underscoring the important role of the NTRK2/TrkB signaling pathway in energy homeostasis (An et al., 2020; Berger et al., 2025). The *NTRK2* gene may explain the severe obesity observed in obesity carriers (Köroğlu et al., 2024).

#### *SH2B1* gene

*SH2B1* encodes an adapter protein that amplifies the signaling pathways of several hormones crucial for energy homeostasis, including leptin and insulin. Mutations in the *SH2B1* gene have shown to have a significant impact on obesity, particularly severe early-onset forms characterized by hyperphagia and insulin resistance. Loss-of-function mutations in the *SH2B1* gene disrupt the leptin signaling pathway in the hypothalamus, causing impaired regulation of appetite and energy expenditure, which also results in increased food intake and rapid weight gain. In other words, these types of mutations contribute to insulin resistance and type 2 diabetes, further complicating the metabolic profile of affected individuals. Behavioral abnormalities such as social isolation and aggression have also been observed in patients with *SH2B1* gene mutations, suggesting a broader role for *SH2B1* gene in neurological function. Mouse models with targeted deletion on the gene *SH2B1* replicate many human phenotypes, such as hyperphagia, obesity, and glucose intolerance, underscoring the critical regulatory role of the gene in metabolism and energy balance (Chermon and Birk, 2024; Doche et al., 2012; Rui, 2014).

Deletions in chromosome 6q16 (including the SIM1 gene), 11p13 (including the BDNF gene), and distal 16p11.2 (including the SH2B1 gene; OMIM #613444) are associated with obesity. (Chung et al., 2021; D’Angelo et al., 2018; Han et al., 2008; Faivre et al., 2002). A study by da Silva et al., showed the same deletion (three genomic deletions at the 16p11.2) in the same region in patients with severe obesity from Brazil. Based on this report, clinical and genetic testing should be carried out to identify patients with the genetic form of severe obesity. This will support specific medical treatment, genetic counselling, and targeted therapeutic intervention (Da Silva Assis et al., 2025).

## BBS-related gene mutations

BBS is a rare autosomal recessive disorder whose main characteristic is obesity. Mutations in several BBS-related genes disrupt the function of the BBSome, a protein complex that is essential for the proper functioning of cilia and that influences energy regulation. Obesity is usually severe and early onset in BBS patients and is often accompanied by hyperphagia and reduced physical activity. Studies in BBS knockout mice, such as those lacking *Bbs2*, *Bbs4*, or *Bbs6*, show increased obesity due to both increased food intake and reduced energy expenditure. It is noteworthy that BBS patients tend to have more visceral fat and altered body composition compared to control subjects with the same BMI. In addition, heterozygous carriers of BBS mutations may have an increased risk of obesity without showing the full syndromal phenotype. These findings show that BBS gene mutations contribute to obesity by impairing central appetite and metabolic regulation via cilia dysfunction, making BBS an important model for understanding syndromic obesity (Benzinou et al., 2006; Guo and Rahmouni, 2011; Li et al., 2024; Zhong et al., 2025).

BBS is a rare autosomal recessive ciliopathy caused by biallelic mutations in 20+ BBS genes (*BBS1*, *BBS2*, *BBS10*, etc.). Disruption of the BBSome-mediated primary cilia function in neurons/adipocytes impairs MC4R trafficking and leptin-melanocortin signaling, causing severe early-onset obesity, hyperphagia, polydactyly, retinitis pigmentosa, and renal/genital anomalies. Mouse models (*Bbs2/4/6* KO) confirm hyperphagia + reduced energy expenditure; heterozygous carriers show increased obesity risk without the full syndrome. Setmelanotide is a known MC4R agonist, which bypasses proximal defects by directly activating MC4R. In a phase 3 trial (NCT03746522), BBS patients showed significant BMI decreases, hunger suppression, and metabolic improvements, earning FDA approval as a first-targeted therapy for BBS obesity, and a 3-year extension confirmed the sustained benefits (stabilization/improvement in weight and QoL) (Haqq et al., 2025; 2022; Yanovski et al., 2024).

## Gene discovery in polygenic obesity

Polygenic obesity results from the additive effects of hundreds to thousands of common genetic variants, each of which confers a small increase in susceptibility to a higher body mass index and obesity (Khera et al., 2019; Loos and Yeo, 2022). GWAS (Genome-Wide Association Study) have reported more than 500 genetic loci associated with enhanced BMI, with the strongest effects observed in variants in genes such as *FTO* and *MC4R*, but each variant alone explains only a small fraction of the phenotypic variance (Hinney et al., 2010; Loos and Yeo, 2022). Genes *SEC16B* and *LINC02702* were also identified in GWAS meta-analyses of obesity traits. In total, more than 1,100 loci have been identified in research on polygenic obesity, underscoring the complex genetic architecture of this trait with many variants, each of which has a small effect; neuroendocrine control of appetite and energy homeostasis are regulated by some of them (Figure 2) (Jo et al., 2024; Smit et al., 2025; Van Uhm et al., 2025). To quantify genetic risk, the different research groups aggregated the effects of these variants into polygenic scores, which show that persons with a high cumulative genetic burden have a significantly increased risk of severe obesity—sometimes even on a scale comparable to the influence of rare monogenic mutations (Khera et al., 2019). In spite of this genetic burden, environmental factors and gene-environment interactions remain crucial in determining whether a person with high polygenic risk will actually develop obesity. Polygenic risk scores (PRS) effectively identify individuals at elevated risk for obesity. They enhance the understanding of obesity’s biological mechanisms. PRS also enables the development of personalized prevention strategies (Khera et al., 2019; Loos and Yeo, 2022).

Table 2 illustrates the complex polygenic architecture of obesity, in which each gene makes a small contribution, as well as the importance of interactions between genes and the environment for the phenotype.

**TABLE 2:** This table shows some of the most important genes that have been repeatedly linked to polygenic obesity, as described in the text and current GWAS research results. There are hundreds to thousands of loci throughout the genome that are associated with polygenic obesity—according to current GWAS meta-analyses, these genes are among the most frequently replicated and biologically plausible. As GWAS samples grow in size and diversity, additional loci will continue to be discovered.

Gene	Full name	Main biological pathway/function	References
*FTO*	Fat mass and obesity-associated	Regulation of energy intake and adipogenesis	Hinney et al. (2010), Merkestein et al. (2015), Loos and Yeo (2022)
*MC4R*	Melanocortin 4 receptor	Appetite regulation, energy balance	Adan et al. (2006), Hinney et al. (2010), ElhamKia et al. (2022), Loos (2025), Hayashi et al. (2025)
*SEC16B*	SEC16 homolog B, endoplasmic reticulum export factor	Lipid metabolism, vesicle trafficking	Jo et al. (2024), Yonekawa et al. (2011)
*LINC02702*	Long intergenic non-protein coding RNA 2702	Regulation of adipogenesis, gene expression	Gluba-Sagr et al. (2024), Jo et al. (2024)
*GNPDA2*	Glucosamine-6-phosphate deaminase 2	Carbohydrate metabolism, energy balance	Jo et al. (2024), Wu et al. (2019)
*BDNF*	Brain-derived neurotrophic factor	CNS appetite and body weight regulation	Cordeira and Rios (2011), Urabe et al. (2013), Jo et al., 2024; Loos (2025)
*ADCY3*	Adenylate cyclase 3	cAMP signaling, energy balance	Pitman et al. (2014), Jo et al. (2024), Khani et al. (2024)
*RALGAPA1*	Ral GTPase activating protein catalytic alpha subunit 1	BMI regulation	Wong et al. (2022)
*IRX3/IRX5*	Iroquois homeobox 3/5	Adipocyte differentiation via FTO locus	Bjune et al. (2025), Wong et al. (2022)
*ZNF259*	Zinc finger protein 259	Metabolic syndrome, possible CVD link	Ellakwa et al. (2024), Jo et al. (2024)

### Polygenic risk score

Polygenic risk scores (PRS) aggregate weighted effects from >1,100 common SNPs identified by GWAS (e.g., GIANT consortium), using methods like PRS-CS/LDpred or clumping + thresholding to account for linkage disequilibrium and effect size uncertainty, yielding a normally distributed score where top deciles confer obesity risk comparable to monogenic mutations. Clinically, PRS predict BMI trajectories from birth, stratify severe obesity risk, and identify lifestyle-responsive high-risk individuals, with healthy behaviors mitigating ∼30–50% of the genetic burden; however, limitations include modest variance explained versus total heritability (40%–70%), European-biased training data causing 50%–70% performance drops in non-Europeans, static scores missing dynamic gene-environment interactions, and weaker midlife prediction than serial BMI monitoring (Choe et al., 2022; Khera et al., 2019).

### Metabolic health

Obesity phenotypes vary widely in metabolic health despite similar BMIs; notably, 10%–30% of individuals are classified as having metabolically healthy obesity (MHO). MHO is characterized by two or fewer metabolic syndrome components—such as normal glucose and lipid levels and an absence of hypertension—in contrast to metabolically unhealthy obesity (MUO), which presents with insulin resistance, dyslipidemia, and increased cardiovascular risk. The MHO associates with polygenic profiles that favor the distribution of subcutaneous fat (*LINC02702* variants), preserving insulin sensitivity, and lower ectopic fat, while the MUO is connected to visceral adiposity and genetic burdens that amplify inflammation/lipotoxicity. Monogenic forms, for example, *MC4R* or *LEP*, typically manifest as MUO due to hyperphagia/energy imbalance disrupting the homeostasis on a metabolic level, though rare MHO-like presentations occur with partial penetrance. PRS stratify MHO/MUO transition risk, showing that high genetic burden accelerates the healthy-to-unhealthy conversion over time (∼30%–50% MHO lose status/decade) (Blüher, 2020).

#### *FTO* gene

Mutations and the most common genetic variants in the *FTO* gene (associated with fat mass and obesity) are associated with an increased risk of obesity through different mechanisms that influence energy balance and fat tissue function. The gene *FTO* is responsible for encoding the RNA demethylase enzyme, in which influences mRNA processing and regulates genes involved in adipogenesis, appetite control, and energy metabolism. Important single nucleotide polymorphisms (SNPs) in the *FTO* gene, such as rs9939609 and rs1421085, have been shown to be associated with a higher risk for high BMI, increased fatty mass, and altered dietary habits, including a tendency to consume energy-dense and high-fat foods. Mechanistically, these variants influence the expression of neighboring regulatory genes such as *IRX3* and *IRX5*, which are responsible for the differentiation of adipocytes from energy-consuming beige cells to energy-storing white fatty cells, alongside reducing mitochondrial thermogenesis and promoting lipid accumulation. Functional studies also show that *FTO* gene variants have a great influence on appetite by modulating hypothalamic circuits and increasing ghrelin expression. Together, these molecular and behavioral effects contribute to a positive energy balance and increased susceptibility to obesity. The *FTO* gene offers potential targets for targeted therapeutic interventions (Kumar et al., 2022; Lan et al., 2020; Poosri et al., 2024; Yang et al., 2017).

#### *MC4R* gene

Mutations in the *MC4R* gene not only influence obesity risk through rare, highly effective variants that cause monogenic obesity (see above) but also interact with polygenic susceptibility to modulate obesity outcomes in broader populations. Recent large-scale studies show that carriers of obesity-associated *MC4R* mutations can exhibit highly diverse phenotypes depending on their polygenic risk score (PRS) for obesity. Individuals with high polygenic risk who also carry *MC4R* mutations have a significantly higher BMI and a higher risk of obesity than mutation carriers with low polygenic risk who remain normal weight or show only slight weight gain. This suggests that the polygenic background can either increase or attenuate the effect of *MC4R* gene mutations. Thus, polygenic susceptibility interacts with *MC4R* gene mutation status and influences the severity and penetrance of obesity. This underscores the complex genetic architecture of this common disease and the importance of integrating information about rare and common variants for personalized risk assessment and treatment strategies (Adamska-Patruno et al., 2021; Chami et al., 2020; Mohn et al., 2025; Wang et al., 2022).

#### *SEC16B* gene

Genetic variants near the *SEC16B* gene have been shown to be linked to polygenic obesity and an increased BMI in several populations, including Japanese and Caucasians. The *SEC16B* genes play a pivotal role in encoding a key protein involved in vesicle transport between the endoplasmic reticulum and the Golgi apparatus, which may have an influence on adipocyte function and energy regulation, although its specific metabolic role has not yet been fully elucidated. The common SNPs such as rs10913469 in *SEC16B* gene show a significant association with obesity risk, likely through the modulation of protein transport on an intracellular level and secretion pathways that influence fat storage and metabolism. These genetic effects add to other obesity-associated loci and thus contribute to the polygenic architecture of obesity. Further functional studies are needed to elucidate the precise biological mechanisms by which *SEC16B* variants influence obesity and metabolic homeostasis (Hotta et al., 2009; Sahibdeen et al., 2018; Shi et al., 2023).

#### *LINC02702* gene

Variants in the gene *LINC02702* have been associated with polygenic obesity, where they influence obesity phenotypes, particularly through their potential regulatory functions during adipogenesis and lipid metabolism. Using a GWAS, the SNP rs486394 within *LINC02702* was identified as significantly associated with increased BMI and metabolic traits related to obesity. Interestingly, the genetic results of this SNP differ between metabolically healthy obesity phenotypes and metabolically unhealthy obesity phenotypes, suggesting that *LINC02702* influences not only obese individuals but also the metabolic health status of obese individuals. Long intergenic non-protein-coding RNAs (lncRNAs) such as *LINC02702* gene are increasingly being recognized for their role in the regulation of gene expression and provide insights into complex genetic interactions underlying polygenic obesity (Jo et al., 2024).

#### *GNPDA2* gene

Variants in the *GNPDA2* gene have been consistently associated with polygenic obesity and an increased BMI in various population studies through GWAS. The *GNPDA2* gene encodes glucosamine-6-phosphate deaminase 2, which is involved in the hexosamine biosynthesis pathway that may influence adipogenesis and glucose metabolism. Studies of gene function suggest that altered *GNPDA2* expression influences lipid accumulation and adipocyte differentiation, thereby contributing to increased fat mass. In addition, *GNPDA2* is expressed in important metabolic tissues such as the hypothalamus, where it may play a role in nutrient sensing and energy balance regulation. The obesity-associated SNP rs10938397 near *GNPDA2* shows a strong correlation with measures of central obesity such as waist circumference and waist-to-height ratio, underscoring its role in obesity distribution. These findings underscore the importance of *GNPDA2* gene as a crucial gene contributing to the polygenic risk for fat mass through modulation of both metabolic and central nervous system signaling pathways (Gutierrez-Aguilar et al., 2021; Wu et al., 2019).

#### *BDNF* gene

Mutations and polymorphisms in the *BDNF* gene are associated with polygenic obesity due to their influence on energy balance, appetite regulation, and neurodevelopment. The common polymorphism Val66Met (SNP rs6265) in the *BDNF* gene alters the intracellular transport and secretion of the protein after maturation, thereby influencing hypothalamic signaling pathways that are critical for satiety and food intake control. Several studies from different research groups have confirmed an association between the *Met* allele and an increased BMI, an increased risk of obesity, and altered metabolic profiles, although the effects differ depending on the population studies and gender. In other words, the regulatory variants are very highly conserved regions of the *BDNF* gene and modulate its expression in the hypothalamus, thus influencing appetite control and energy homeostasis. The functional impact of these *BDNF* variants can also be influenced by other factors such as environmental factors, diet, and physical activity, which further contribute to the complex genetic architecture of polygenic obesity (Honarmand et al., 2021; McEwan et al., 2024; Miksza et al., 2023).

#### *ADCY3* gene3

Mutations in the *ADCY3* gene have been associated with polygenic obesity due to their influence on neural pathways regulating appetite and energy homeostasis. *ADCY3* encodes adenylate cyclase 3, an enzyme that is primarily expressed in the primary cilia of hypothalamic neurons, where it catalyzes the production of cyclic AMP (cAMP), an important secondary messenger for appetite regulation and metabolic control. Variants of the *ADCY3* gene that impair its function, especially homozygous loss-of-function mutations, have the ability to disrupt cAMP signaling pathways, which lead to excessive eating, severe obesity beginning in early childhood, insulin resistance, and delays in neurological development. Animal models confirm that loss of *ADCY3* function disrupts leptin-melanocortin signaling pathways, particularly through its colocalization and interaction with MC4R in hypothalamic neurons, which are essential for normal body weight regulation. Moreover, certain *ADCY3* gene polymorphisms are associated with obesity risk in various human populations, confirming its relation to polygenic obesity susceptibility. These findings emphasize how important *ADCY3* is as a crucial genetic factor influencing obesity via central nervous system pathways that control feeding behavior and energy balance (Mohammed et al., 2024; Toumba et al., 2021).

#### *RALGAPA1* gene

The gene mutations found in the *RALGAPA1* gene have been associated with polygenic obesity due to their pivotal role in intracellular signaling pathways that regulate energy balance and obesity. *RALGAPA1* is responsible for encoding a GTPase-activating protein involved in the regulation of the Ral signaling pathway, which influences different cellular processes such as vesicle transport and cytoskeletal dynamics. Although direct functional studies on the role of *RALGAPA1* in obesity are limited, the GWAS have found variants near or within the gene that correlate with increased BMI and obesity risk. These variants likely contribute with small additive effects within the polygenic architecture of obesity and influence fat accumulation and metabolic regulation. Research findings suggest that *RALGAPA1* may interact with other obesity-associated genes to modulate hypothalamic control of appetite and peripheral metabolism, thereby contributing to the complex genetic predisposition observed in general obesity (Hinney and Hebebr, 2008).

#### *IRX3*/*IRX5* gene

Mutations in the *IRX3* and *IRX5* genes have a significant influence on polygenic obesity, primarily through their regulatory effects on energy homeostasis, adipose tissue function, and hypothalamic appetite control. These genes are located near the *FTO* locus, the strongest genetic risk region for polygenic obesity, and the expression of *IRX3* and *IRX5* is modulated by obesity-associated variants within *FTO* through extensive enhancer-promoter interactions. The increasing expression of *IRX3* and *IRX5* genes in adipocytes lead to reduced thermogenesis of adipose tissue by promoting the storage of white fat over the formation of beige fat, thereby reducing energy expenditure. Moreover, in mouse models, the inhibition of *Irx3* or *Irx5* genes leads to lean phenotypes characterized by increased basal metabolic rate, increased adipose browning, decreased food intake, and a resistance to diet-induced obesity. The expression of these genes is also increased in hypothalamic neurons, suggesting their role in the central regulation of feeding behavior. These coordinated peripheral and central effects position *IRX3* and *IRX5* as important mediators of *FTO*-associated obesity risk and contribute to the complex polygenic architecture of obesity (Dou et al., 2021; Sobreira et al., 2021; Son et al., 2022).

#### *ZNF259* gene mutation

Variants in the *ZNF259* gene are associated with polygenic obesity because they influence lipid metabolism and energy homeostasis. The gene *ZNF259* is responsible for encoding a zinc finger protein that participates in regulating lipid profile and cell metabolism. Different genetic studies have identified polymorphisms within or near *ZNF259* that are correlated with an increased BMI and dyslipidemia, both of which are risk factors for obesity. These variants contribute to obesity risk by modulating metabolic pathways that influence fat storage, insulin sensitivity, and systemic inflammation. Although the exact mechanisms are still being investigated, the cumulative effect of *ZNF259* gene mutations in a polygenic context underscores its role as one of several genes with moderate effects on obesity susceptibility and metabolic dysregulation in affected individuals (Parra et al., 2017; Vázquez-Moreno et al., 2021).

## Weight loss medications: scientific findings and clinical studies

The development of pharmacological agents for weight loss has intensified over the past decade, with several classes of anti-obesity medications targeting metabolic, hormonal, and appetite-regulating signaling pathways. Key breakthroughs include GLP-1 receptor agonists, dual incretin agonists, and newer, multi-hormonal therapies for obese patients that have been shown to demonstrate significant and sustained weight loss in both clinical trials and real-world practice.

## Important classes of active substances and clinical study results

### GLP-1 receptor agonists

- Semaglutide: A GLP-1 receptor agonist administered once weekly results in significant and sustained weight loss in overweight and obese patients. In the randomized controlled trials STEP 5, SELECT, and STEP 1, participants who received 2.4 mg semaglutide subcutaneously lost 10%–15% or more of their body weight over a period of 68–104 weeks, which was significantly more than with placebo and behavioral interventions. At week 104, an estimated 77% of participants achieved a weight loss of ≥5% and over a third achieved a reduction of 20% or more. The most common side effects include mild to moderate gastrointestinal symptoms (clinicaltrials.gov/study/NCT03548935) (Garvey et al., 2022; Ghusn et al., 2022; Ryan et al., 2024).
- New oral agents: Oral semaglutide and other oral GLP-1 formulations (VK2735, amycretin) are currently being evaluated and show promising initial clinical results, which may improve accessibility.

### Dual incretin receptor agonist

- Tirzepatide: This once-weekly dual agonist of GLP-1 and glucose-dependent insulinotropic polypeptide (GIP) is associated with even greater weight loss. In randomized studies, tirzepatide as an add-on to an intensive lifestyle program over 72 weeks resulted in an additional average weight loss of 18.4% (compared to a 2.5% increase with placebo). Meta-analyses confirm its superiority over placebo and GLP-1 therapy alone with consistent efficacy in patients with and without diabetes, although higher rates of gastrointestinal side effects are reported (ClinicalTrials.gov–NCT04660643) (Aryee et al., 2025; Lin et al., 2023; Wadden et al., 2023).

### Triple and multi-hormonal agonist

- Retatrutide: This novel active ingredient, which is administered once a week, acts on GLP-1, GIP, and glucagon receptors. A 48-week double-blind study found that 64%–100% of participants treated with retatrutide achieved ≥5% weight loss, and up to 26% of subjects lost 30% or more of their baseline weight. Using retatrutide is also shown to improve several cardiometabolic parameters and it demonstrates a favorable safety profile with comparable rates of adverse events to placebo (Jastreboff et al., 2023; Naeem et al., 2024).

### Oral GLP-1 agonists

- Orforglipron: A once-daily oral non-peptide GLP-1 receptor agonist has achieved significant weight loss in both Phase 1 and Phase 3 clinical trials. In the ACHIEVE-1 trial, adults with diabetes experienced an average weight reduction of about 16 pounds, corresponding to roughly 7.9% of their body weight, after 40 weeks of treatment with the highest dosage. In initial studies with healthy subjects, weight loss of ∼5.4 kg was observed after 4 weeks, with gastrointestinal events being the most common side effects (Ma et al., 2024; Pratt et al., 2023).

The addition of other active ingredients such as oral GLP-1 analogues and experimental combination therapies is currently being investigated and is expected to improve efficacy, ease of use, and accessibility in the coming years (Dolgin, 2025).

## Comparative efficacy and real-world observations

In randomized controlled trials, semaglutide, tirzepatide, and retatrutide consistently result in double-digit percentage total weight loss, significantly outperforming previous obesity pharmacotherapy. Direct comparison studies and sequential studies demonstrate the superiority of dual and triple agonists (such as tirzepatide and retatrutide) over single-hormone therapies, suggesting a trend toward combination mechanisms for more effective weight loss (Lin et al., 2023; Naeem et al., 2024; Wadden et al., 2023).

Practical studies show a slightly lower average weight loss due to discontinuation of treatment and variable dosing, but significant benefits continue to be achieved when the therapy is adhered to (Tzoulis et al., 2024; Weiss et al., 2022). Safety profiles are generally favorable, with gastrointestinal side effects (nausea, vomiting, and diarrhea) being the most common. Serious adverse events are rare, and overall tolerability is high for the leading agents (Table 3) (Garvey et al., 2022; Jastreboff et al., 2023).

**TABLE 3:** Summary of clinical trial results for leading weight loss anti-obesity medications.

Drug	Mechanism	Formulation	Trial duration	Mean weight loss	% ≥5% body weight loss	Key trials
Semaglutide	GLP-1 RA	Weekly inject	68–208 weeks	10%–15% (Garvey et al., 2022; Ghusn et al., 2022; Ryan et al., 2024)	67%–77% (Garvey et al., 2022; Ryan et al., 2024)	STEP, SELECT
Tirzepatide	GLP-1/GIP dual agonist	Weekly inject	72 weeks	Up to 18.4% (Lin et al., 2023; Wadden et al., 2023)	87% (Lin et al., 2023; Wadden et al., 2023)	SURMOUNT
Retatrutide	GLP-1/GIP/glucagon triple	Weekly inject	48 weeks	Up to 24% (Jastreboff et al., 2023; Naeem et al., 2024)	64%–100% (Jastreboff et al., 2023; Naeem et al., 2024)	Phase 2, 3
Orforglipron	Oral GLP-1 RA	Daily pill	4–40 weeks	5%–8% (Ma et al., 2024; Pratt et al., 2023)	Not detailed	ACHIEVE-1

Modern anti-obesity medications—such as semaglutide, tirzepatide, retatrutide, and orforglipron—mark a paradigm shift in the pharmacological treatment of obesity. They enable significant weight loss in conjunction with improvements in metabolic risk factors, as demonstrated by numerous large-scale clinical trials. These anti-obesity medications pave the way for more accessible, convenient options (including once-daily tablets) and combination therapies, although their effectiveness in practice depends on long-term adherence and individual patient selection (Dolgin, 2025; Garvey et al., 2022; Jastreboff et al., 2023; Naeem et al., 2024; Ryan et al., 2024; Wadden et al., 2023).

## Links between weight loss anti-obesity medications and genetic mutations in obesity

### Genetic mutations associated with obesity

As discussed, obesity has a strong genetic component, with both rare, highly effective mutations (monogenic forms) and several common genetic variants contributing to the risk. Key genes linked to severe or early-onset obesity include *MC4R, LEP, LEPR, POMC, PCSK1,* and *FTO*, all of which play pivotal roles in energy regulation and metabolic processes. The gene *MC4R* is the most commonly mutated gene in monogenic obesity, and variants of the *FTO* gene are closely associated with an increased body mass index and frequent obesity in the general population (Choquet and Meyre, 2011; González Jiménez, 2011; Herrera et al., 2011; Loos and Yeo, 2022; Mahmoud et al., 2022). In patients with extremely high BMI (e.g., >40 kg/m^2^), gene mutations that influence these signaling pathways are relatively common (Al-Humadi et al., 2023).

The impact of genetic mutations on the effectiveness of weight loss medications has been demonstrated, with variations in genes influencing the response and outcomes of treatment.

### GLP-1 receptor agonists (semaglutide, liraglutide, tirzepatide, etc.)

The leading anti-obesity medications (GLP-1 receptor agonists, dual/triple incretin agonists, oral GLP-1 tablets) have been studied for their effectiveness in different genetic backgrounds:

- Recent multi-biobank studies from different research groups involving more than 10,000 individuals have found that common genetic variants associated with obesity, including those in *MC4R*, *FTO*, and polygenic obesity risk scores, had no clinically significant impact on the efficacy of GLP-1 receptor agonists for weight loss. These anti-obesity medications, such as semaglutide and tirzepatide, achieve similar levels of weight loss irrespective of genetic background, suggesting that patients with high-risk mutations for obesity benefit to a similar extent as patients without such variants (German et al., 2025; Wong et al., 2025).
- SNPs of the *GLP1R* gene: Other pharmacogenetically relevant loci have presented only minor and inconsistent associations with weight loss in smaller studies, suggesting that the known mutations of obesity genes cause only minor differences in drug response (BouSaba et al., 2023; Dawed et al., 2023; German et al., 2025).

## Influence of genetic predisposition

Some research groups have suggested that a lower overall genetic predisposition to obesity may be correlated with slightly greater weight loss under GLP-1 therapy, but the variations observed are generally small and are not considered to limit the treatment benefit for individuals at high genetic risk (Levy et al., 2024). Genetic variability plays a more pivotal role in the development of obesity than in the effectiveness of pharmacological interventions for weight loss (Cifuentes et al., 2025; German et al., 2025; Wong et al., 2025) (Table 4).

**TABLE 4:** Obesity genes and pharmacologic response.

Main genes involved	Associated with obesity	Impact on drug response
*MC4R, LEP, LEPR, FTO*	Strong effect, monogenic/polygenic forms (Loos and Yeo, 2022; Mahmoud et al., 2022; Choquet and Meyre, 2011; Herrera et al., 2011; González Jiménez, 2011; Tirthani et al., 2025)	Little to no clinically significant difference in weight loss with GLP-1 anti-obesity medications Wong et al., 2025; Levy et al., 2024)
*GLP1R, CNR1*	Minor influence on specific responses (BouSaba et al., 2023; Dawed et al., 2023)	No consistent or strong effect detected

### Clinical implications

The most effective and commonly used weight loss anti-obesity medications—GLP-1 receptor agonists (injectable and oral) and dual and triple incretin agonists—show broad efficacy regardless of individual genetic risk for obesity. Current findings support prescribing these anti-obesity medications to people with a high genetic predisposition to obesity, as their weight loss results are comparable to those of individuals without significant obesity-associated mutations. While personalized genetic testing can identify rare cases in which alternative treatments such as other anti-obesity medications or bariatric surgery may offer additional benefits, for the majority of patients, genetic mutations do not significantly influence the likelihood of successful weight loss through pharmacotherapy (Cifuentes et al., 2025; German et al., 2025; Wong et al., 2025).

## Discussion

Obesity is a complex clinical challenge caused by environmental influences and genetic predisposition, which has been uncovered by intensive genetic research. Monogenic obesity results from rare, highly penetrant mutations that primarily affect neuroendocrine regulators such as *MC4R*, *LEP*, *LEPR*, *POMC*, and *PCSK1* and syndromic genes, including Bardet-Biedl variants. These mutations show severe, early-onset phenotypes and define discrete clinical entities that are suitable for targeted interventions such as MC4R agonists. Conversely, polygenic obesity arises from the additive effect of numerous common variants—over 1,100 loci have been identified in GWAS—each of which has a moderate phenotypic influence. The important polygenic genes such as *FTO*, *MC4R*, *SEC16B*, and *LINC02702* together modulate appetite, energy balance, and adipogenesis via overlapping neuroendocrine signaling pathways, illustrating a common biological framework with monogenic forms.

The intersection of monogenic and polygenic obesity signalling pathways highlights the critical role of neuroendocrine signalling networks, especially the leptin-melanocortin pathway, in the development of obesity. Importantly, gene-environment interactions shape the phenotypic spectrum, which complicates risk prediction but offers intervention loci. Polygenic risk scores are powerful predictive tools but need to be validated in independent populations.

Pharmacological advances, particularly GLP-1 receptor agonists and dual/triple incretin therapies, have shown efficacy in weight decrease irrespective of genetic background and offer promising precision treatments for obesity. Genetic background has minimal influence on response to therapy, suggesting broad applicability. However, personalized medical approaches that leverage genetic diagnostics could optimize patient selection and identify new therapeutic targets (Ko et al., 2025).

The integration of genetic examination into routine care remains a challenge due to cost, complexity, and incomplete understanding of all relevant loci. The growth of polygenic risk models and the unraveling of mechanistic signaling pathways will improve the precision of prevention and treatment. Ultimately, multidisciplinary approaches combining genomics, lifestyle changes, and new therapeutics will be crucial in combating the obesity epidemic.

### Conclusion

This overview highlights the complex genetic architecture of obesity, ranging from rare monogenic mutations in important neuroendocrine genes to widespread common variants that contribute to polygenic risk. The integration of genetic insights has improved understanding of biological mechanisms and advanced the development of effective pharmacotherapies, marking a paradigm shift toward precision medicine for obesity. Although current anti-obesity medications are effective regardless of genetic background, genetic risk stratification using polygenic scores promises individualized prevention and tailored treatment strategies. Further research on gene-environment interactions, more comprehensive genetic screening, and functional characterization of obesity-associated loci are essential. Future efforts must focus on translating genomic discoveries into accessible clinical tools to improve obesity treatment, alleviate comorbidities, and improve treatment outcomes for patients worldwide.

This review elucidates obesity’s genetic architecture, contrasting rare monogenic forms (e.g., *MC4R*, *LEP*, and *POMC* gene mutations in leptin-melanocortin signalling pathways) with common polygenic forms (like *FTO* and *SEC16B*). Key findings highlight shared neuroendocrine mechanisms, gene-environment interactions, polygenic risk scores for stratification, and GLP-1/incretin agonists’ efficacy across genetic backgrounds.​

These align with objectives by advancing precision pharmacotherapy: genetic insights enable risk prediction and tailored interventions despite challenges in screening and interactions. Future priorities include expanded genomics, functional studies, and clinical translation for optimized outcomes.

## Footnotes

1
https://www.who.int/news-room/fact-sheets/detail/obesity-and-overweight

## References

[B1] Adamska-Patruno E. Bauer W. Bielska D. Fiedorczuk J. Moroz M. Krasowska U. (2021). An association between diet and MC4R genetic polymorphism, in relation to obesity and metabolic parameters—A cross sectional population-based study. IJMS 22, 12044. 10.3390/ijms222112044 34769477 PMC8584592

[B2] Adan R. A. H. Tiesjema B. Hillebrand J. J. G. La Fleur S. E. Kas M. J. H. De Krom M. (2006). The MC4 receptor and control of appetite. Br. J Pharmacol. 149, 815–827. 10.1038/sj.bjp.0706929 17043670 PMC2014686

[B3] Al-Humadi A. W. Alabduljabbar K. Alsaqaaby M. S. Talaee H. Le Roux C. W. (2023). Obesity characteristics are poor predictors of genetic mutations associated with obesity. JCM 12, 6396. 10.3390/jcm12196396 37835041 PMC10573901

[B4] Alhamas A. R. Alhashem A. M. Alasmari A. Faqeih E. A. (2022). NTRK2-Related obesity, hyperphagia, and developmental delay: case report. JBCJ 5, 48–52. 10.24911/JBCGenetics/183-1665949143

[B5] An J. J. Kinney C. E. Tan J.-W. Liao G.-Y. Kremer E. J. Xu B. (2020). TrkB-expressing paraventricular hypothalamic neurons suppress appetite through multiple neurocircuits. Nat. Commun. 11, 1729. 10.1038/s41467-020-15537-w 32265438 PMC7138837

[B6] Aryee E. K. Zhang S. Selvin E. Fang M. (2025). Prevalence of obesity with and without confirmation of excess adiposity among US adults. JAMA 333, 1726–1728. 10.1001/jama.2025.2704 40244602 PMC12006908

[B7] Benzinou M. Walley A. Lobbens S. Charles M.-A. Jouret B. Fumeron F. (2006). Bardet-biedl syndrome gene variants are associated with both childhood and adult common obesity in French Caucasians. Diabetes 55, 2876–2882. 10.2337/db06-0337 17003356

[B8] Berger E. Jauss R.-T. Ranells J. D. Zonic E. Von Wintzingerode L. Wilson A. (2025). Upregulation *versus* loss of function of NTRK2 in 44 affected individuals leads to 2 distinct neurodevelopmental disorders. Genet. Med. 27, 101326. 10.1016/j.gim.2024.101326 39540377

[B9] Bjune J.-I. Laber S. Lawrence-Archer L. Nothnagel P. M. C. Yamada S. Zhao X. (2025). IRX3 controls a SUMOylation-dependent differentiation switch in adipocyte precursor cells. Nat. Commun. 16, 7248. 10.1038/s41467-025-62361-1 40769964 PMC12328774

[B10] Blüher M. (2020). Metabolically healthy obesity. Endocr. Rev. 41, bnaa004. 10.1210/endrev/bnaa004 32128581 PMC7098708

[B11] Bonnefond A. Raimondo A. Stutzmann F. Ghoussaini M. Ramachandrappa S. Bersten D. C. (2013). Loss-of-function mutations in SIM1 contribute to obesity and prader-willi–like features. J. Clin. Invest. 123, 3037–3041. 10.1172/JCI68035 23778136 PMC3696559

[B12] BouSaba J. Vosoughi K. Dilmaghani S. Prokop L. J. Camilleri M. (2023). Pharmacogenetic interactions of medications administered for weight loss in adults: a systematic review and meta-analysis. Pharmacogenomics 24, 283–295. 10.2217/pgs-2022-0192 36999540 PMC10152409

[B13] Çetinkaya S. Güran T. Kurnaz E. Keskin M. Sağsak E. Savaş Erdeve S. (2018). A patient with proopiomelanocortin deficiency: an increasingly important diagnosis to make. Jcrpe 10, 68–73. 10.4274/jcrpe.4638 28739551 PMC5838375

[B14] Chami N. Preuss M. Walker R. W. Moscati A. Loos R. J. F. (2020). The role of polygenic susceptibility to obesity among carriers of pathogenic mutations in MC4R in the UK Biobank population. PLoS Med. 17, e1003196. 10.1371/journal.pmed.1003196 32692746 PMC7373259

[B15] Chaves C. Kay T. Anselmo J. (2022). Early onset obesity due to a mutation in the human leptin receptor gene. Endocrinol. Diabetes and Metabolism Case Rep. 2022, 21–0124. 10.1530/EDM-21-0124 36001025 PMC9422261

[B16] Chermon D. Birk R. (2024). Gene-environment interactions significantly alter the obesity risk of SH2B1 rs7498665 carriers. J. Obes. and Metabolic Syndrome 33, 251–260. 10.7570/jomes23066 39098052 PMC11443330

[B17] Choe E. K. Shivakumar M. Lee S. M. Verma A. Kim D. (2022). Dissecting the clinical relevance of polygenic risk score for obesity—a cross-sectional, longitudinal analysis. Int. J. Obes. 46, 1686–1693. 10.1038/s41366-022-01168-2 35752651 PMC10362905

[B18] Choquet H. Meyre D. (2011). Genetics of obesity: what have we learned? CG 12, 169–179. 10.2174/138920211795677895 22043165 PMC3137002

[B19] Chung W. K. (2012). An overview of mongenic and syndromic obesities in humans. Pediatr. Blood and Cancer 58, 122–128. 10.1002/pbc.23372 21994130 PMC3215910

[B20] Chung W. K. Roberts T. P. Sherr E. H. Snyder L. G. Spiro J. E. (2021). 16p11.2 deletion syndrome. Curr. Opin. Genet. and Dev. 68, 49–56. 10.1016/j.gde.2021.01.011 33667823 PMC10256135

[B21] Cifuentes L. Anazco D. O’Connor T. Hurtado M. D. Ghusn W. Campos A. (2025). Genetic and physiological insights into satiation variability predict responses to obesity treatment. Cell Metab. 37, 1655–1666.e5. 10.1016/j.cmet.2025.05.008 40482646 PMC12236147

[B22] Collet T.-H. Schwitzgebel V. (2024). Exploring the therapeutic potential of precision medicine in rare genetic obesity disorders: A scientific perspective. Front. Nutr. 11, 1509994. 10.3389/fnut.2024.1509994 39777073 PMC11705004

[B23] Cordeira J. Rios M. (2011). Weighing in the role of BDNF in the central control of eating behavior. Mol. Neurobiol. 44, 441–448. 10.1007/s12035-011-8212-2 22012072 PMC3235948

[B24] Da Silva Assis I. S. Salum K. C. R. Felício R. D. F. M. Palhinha L. De Medeiros Abreu G. Silva T. (2025). Genomic deletions on 16p11.2 associated with severe obesity in Brazil. Front. Endocrinol. 15, 1495534. 10.3389/fendo.2024.1495534 39897959 PMC11781945

[B25] Dawed A. Y. Mari A. Brown A. McDonald T. J. Li L. Wang S. (2023). Pharmacogenomics of GLP-1 receptor agonists: A genome-wide analysis of observational data and large randomised controlled trials. Lancet Diabetes and Endocrinol. 11, 33–41. 10.1016/S2213-8587(22)00340-0 36528349

[B26] Dayal D. Seetharaman K. Panigrahi I. Muthuvel B. Agarwal A. (2018). Severe early onset obesity due to a novel missense mutation in exon 3 of the leptin gene in an infant from Northwest India. Jcrpe 10, 274–278. 10.4274/jcrpe.5501 29217499 PMC6083471

[B27] Doche M. E. Bochukova E. G. Su H.-W. Pearce L. R. Keogh J. M. Henning E. (2012). Human SH2B1 mutations are associated with maladaptive behaviors and obesity. J. Clin. Invest. 122, 4732–4736. 10.1172/JCI62696 23160192 PMC3533535

[B28] Dolgin E. (2025). Dozens of new obesity drugs are coming: These are the ones to watch. Nature 638, 308–310. 10.1038/d41586-025-00404-9 39939789

[B29] Dou Z. Son J. E. Hui C. (2021). Irx3 and Irx5 - Novel regulatory factors of postnatal hypothalamic neurogenesis. Front. Neurosci. 15, 763856. 10.3389/fnins.2021.763856 34795556 PMC8593166

[B30] Doulla M. McIntyre A. D. Hegele R. A. Gallego P. H. (2014). A novel MC4R mutation associated with childhood-onset obesity: a case report. Paediatr. and Child Health 19, 515–518. 10.1093/pch/19.10.515 25587224 PMC4276379

[B31] Dubern B. Lubrano-Berthelier C. Mencarelli M. Ersoy B. Frelut M.-L. Bouglé D. (2008). Mutational analysis of the pro-opiomelanocortin gene in French obese children led to the identification of a novel deleterious heterozygous mutation located in the α-Melanocyte stimulating hormone domain. Pediatr. Res. 63, 211–216. 10.1203/PDR.0b013e31815ed62b 18091355

[B32] D’Angelo C. S. Varela M. C. De Castro C. I. E. Otto P. A. Perez A. B. A. Lourenço C. M. (2018). Chromosomal microarray analysis in the genetic evaluation of 279 patients with syndromic obesity. Mol. Cytogenet 11, 14. 10.1186/s13039-018-0363-7 29441128 PMC5800070

[B33] ElhamKia M. Setayesh L. Yarizadeh H. Pooyan S. Veisy Z. Aghamohammadi V. (2022). The interaction between dietary total antioxidant capacity and MC4R gene and HOMA-IR in metabolically healthy and unhealthy overweight and Obese women. Nutr. Metab. Insights 15, 11786388221105984. 10.1177/11786388221105984 35734030 PMC9208029

[B34] Elks C. E. Den Hoed M. Zhao J. H. Sharp S. J. Wareham N. J. Loos R. J. F. (2012). Variability in the heritability of body mass index: a systematic review and meta-regression. Front. Endocrin. 3, 29. 10.3389/fendo.2012.00029 22645519 PMC3355836

[B35] Ellakwa D.E.-S. Amr K. S. Zaki M. E. Refeat M. Banksle H. M. (2024). Zinc finger 259 gene polymorphisms in Egyptian patients with metabolic syndrome and its association with dyslipidemia. Ir. J. Med. Sci. 193, 2313–2323. 10.1007/s11845-024-03752-z 38985417

[B36] Faivre L. Cormier-Daire V. Lapierre J. M. Colleaux L. Jacquemont S. Geneviéve D. (2002). Deletion of the *SIM1* gene (6q16.2) in a patient with a prader-willi-like phenotype. J. Med. Genet. 39, 594–596. 10.1136/jmg.39.8.594 12161602 PMC1735217

[B37] Farooqi S. (2023). Obesity and thinness: Insights from genetics. Phil. Trans. R. Soc. B 378, 20220205. 10.1098/rstb.2022.0205 37661743 PMC10475868

[B38] Fischer-Posovszky P. Von Schnurbein J. Moepps B. Lahr G. Strauss G. Barth T. F. (2010). A new missense mutation in the leptin gene causes mild obesity and hypogonadism without affecting T cell responsiveness. J. Clin. Endocrinol. and Metabolism 95, 2836–2840. 10.1210/jc.2009-2466 20382689

[B39] Garvey W. T. Batterham R. L. Bhatta M. Buscemi S. Christensen L. N. Frias J. P. (2022). Two-year effects of semaglutide in adults with overweight or obesity: The STEP 5 trial. Nat. Med. 28, 2083–2091. 10.1038/s41591-022-02026-4 36216945 PMC9556320

[B40] German J. Cordioli M. Tozzo V. Urbut S. Arumäe K. Smit R. A. J. (2025). Association between plausible genetic factors and weight loss from GLP1-RA and bariatric surgery. Nat. Med. 31, 2269–2276. 10.1038/s41591-025-03645-3 40251273 PMC12283387

[B41] Ghusn W. De La Rosa A. Sacoto D. Cifuentes L. Campos A. Feris F. (2022). Weight loss outcomes associated with semaglutide treatment for patients with overweight or obesity. JAMA Netw. Open 5, e2231982. 10.1001/jamanetworkopen.2022.31982 36121652 PMC9486455

[B42] Giannopoulou E. Z. Zorn S. Schirmer M. Brandt-Heunemann S. Schnurbein J. V. Nestoris C. (2026). Monogenic obesity due to MC4R deficiency: Lessons from a multigenerational case. Mol. Cell Pediatr. 13, 3. 10.1186/s40348-025-00214-z 41489710 PMC12770134

[B43] Gluba-Sagr A. Franczyk B. Rysz-Górzyńska A. Olszewski R. Rysz J. (2024). The role of selected lncRNAs in lipid metabolism and cardiovascular disease risk. IJMS 25, 9244. 10.3390/ijms25179244 39273193 PMC11395304

[B44] González Jiménez E. (2011). Genes and obesity: a cause and effect relationship. Endocrinol. Y Nutr. English Ed. 58, 492–496. 10.1016/j.endoen.2011.06.005 21917535

[B45] Gray J. Yeo G. Hung C. Keogh J. Clayton P. Banerjee K. (2007). Functional characterization of human NTRK2 mutations identified in patients with severe early-onset obesity. Int. J. Obes. 31, 359–364. 10.1038/sj.ijo.0803390 16702999

[B46] Guo D.-F. Rahmouni K. (2011). Molecular basis of the obesity associated with Bardet–Biedl syndrome. Trends Endocrinol. and Metabolism S1043276011000361, 286–293. 10.1016/j.tem.2011.02.009 21514177 PMC3130119

[B47] Gutierrez-Aguilar R. Grayson B. E. Kim D.-H. Yalamanchili S. Calcagno M. L. Woods S. C. (2021). CNS GNPDA2 does not control appetite, but regulates glucose homeostasis. Front. Nutr. 8, 787470. 10.3389/fnut.2021.787470 34912841 PMC8666973

[B48] Han J. C. Liu Q.-R. Jones M. Levinn R. L. Menzie C. M. Jefferson-George K. S. (2008). Brain-derived neurotrophic factor and obesity in the WAGR syndrome. N. Engl. J. Med. 359, 918–927. 10.1056/NEJMoa0801119 18753648 PMC2553704

[B49] Hanssen R. Auwerx C. Jõeloo M. Sadler M. C. Henning E. Keogh J. (2023). Chromosomal deletions on 16p11.2 encompassing SH2B1 are associated with accelerated metabolic disease. Cell Rep. Med. 4, 101155. 10.1016/j.xcrm.2023.101155 37586323 PMC10439272

[B50] Haqq A. M. Chung W. K. Dollfus H. Haws R. M. Martos-Moreno G. Á. Poitou C. (2022). Efficacy and safety of setmelanotide, a melanocortin-4 receptor agonist, in patients with Bardet-Biedl syndrome and Alström syndrome: A multicentre, randomised, double-blind, placebo-controlled, phase 3 trial with an open-label period. Lancet Diabetes and Endocrinol. 10, 859–868. 10.1016/S2213-8587(22)00277-7 36356613 PMC9847480

[B51] Haqq A. M. Poitou C. Chung W. K. Forsythe E. Conroy R. Dollfus H. (2025). Impact of setmelanotide on metabolic syndrome risk in patients with Bardet-Biedl syndrome. J. Clin. Endocrinol. and Metabolism 110, e3271–e3282. 10.1210/clinem/dgaf079 39919037 PMC12448622

[B52] Hayashi T. Kumamoto K. Kobayashi T. Hou X. Nagao S. Harada N. (2025). Estrogen synthesized in the central nervous system enhances MC 4 R expression and reduces food intake. FEBS J. 292, 3900–3909. 10.1111/febs.17426 39967403 PMC12326884

[B53] Herrera B. M. Keildson S. Lindgren C. M. (2011). Genetics and epigenetics of obesity. Maturitas 69, 41–49. 10.1016/j.maturitas.2011.02.018 21466928 PMC3213306

[B54] Hinney A. Hebebr J. (2008). Polygenic obesity in humans. Obes. Facts 1, 35–42. 10.1159/000113935 20054160 PMC6444787

[B55] Hinney A. Vogel C. I. G. Hebebrand J. (2010). From monogenic to polygenic obesity: Recent advances. Eur. Child. Adolesc. Psychiatry 19, 297–310. 10.1007/s00787-010-0096-6 20127379 PMC2839509

[B56] Honarmand H. Bonyadi M. Rafat A. Mahdavi R. Aliasghari F. (2021). Association study of the BDNF gene polymorphism (G196A) with overweight/obesity among women from Northwest of Iran. Egypt J. Med. Hum. Genet. 22, 7. 10.1186/s43042-020-00130-z

[B57] Hotta K. Nakamura M. Nakamura T. Matsuo T. Nakata Y. Kamohara S. (2009). Association between obesity and polymorphisms in SEC16B, TMEM18, GNPDA2, BDNF, FAIM2 and MC4R in a Japanese population. J. Hum. Genet. 54, 727–731. 10.1038/jhg.2009.106 19851340

[B58] Huvenne H. Dubern B. Clément K. Poitou C. (2016). Rare genetic forms of obesity: clinical approach and current treatments in 2016. Obes. Facts 9, 158–173. 10.1159/000445061 27241181 PMC5644891

[B59] Imangaliyeva A. Sikhayeva N. Bolatov A. Utupov T. Romanova A. Akhmetollayev I. (2025). Genetic insights into severe obesity: a case study of MC4R variant identification and clinical implications. Genes 16, 508. 10.3390/genes16050508 40428329 PMC12111737

[B60] Jastreboff A. M. Kaplan L. M. Frías J. P. Wu Q. Du Y. Gurbuz S. (2023). Triple–hormone-receptor agonist retatrutide for obesity — a phase 2 trial. N. Engl. J. Med. 389, 514–526. 10.1056/nejmoa2301972 37366315

[B61] Jo J. Ha N. Ji Y. Do A. Seo J. H. Oh B. (2024). Genetic determinants of obesity in Korean populations: exploring genome-wide associations and polygenic risk scores. Briefings Bioinforma. 25, bbae389. 10.1093/bib/bbae389 39207728 PMC11359806

[B62] Jr J. L. (2000). Profound obesity associated with a balanced translocation that disrupts the SIM1 gene. Hum. Mol. Genet. 9, 101–108. 10.1093/hmg/9.1.101 10587584

[B63] Keith S. W. Redden D. T. Katzmarzyk P. T. Boggiano M. M. Hanlon E. C. Benca R. M. (2006). Putative contributors to the secular increase in obesity: exploring the roads less traveled. Int. J. Obes. 30, 1585–1594. 10.1038/sj.ijo.0803326 16801930

[B64] Khani S. Topel H. Kardinal R. Tavanez A. R. Josephrajan A. Larsen B. D. M. (2024). Cold-induced expression of a truncated adenylyl cyclase 3 acts as rheostat to brown fat function. Nat. Metab. 6, 1053–1075. 10.1038/s42255-024-01033-8 38684889 PMC11971047

[B65] Khera A. V. Chaffin M. Wade K. H. Zahid S. Brancale J. Xia R. (2019). Polygenic prediction of weight and obesity trajectories from birth to adulthood. Cell 177, 587–596.e9. 10.1016/j.cell.2019.03.028 31002795 PMC6661115

[B66] Kleinendorst L. Abawi O. Van Der Kamp H. J. Alders M. Meijers-Heijboer H. E. J. Van Rossum E. F. C. (2020). Leptin receptor deficiency: a systematic literature review and prevalence estimation based on population genetics. Eur. J. Endocrinol. 182, 47–56. 10.1530/EJE-19-0678 31658438

[B67] Ko T. Y. L. McGillicuddy D. Al-Humadi A. W. Al-Najim W. Miras A. D. Pournaras D. J. (2025). The role of genetic testing in obesity care: the patient perspective. Obes. Endocrinol. 1, 1–9. 10.1093/obendo/wjaf001

[B68] Köroğlu Ç. Traurig M. Muller Y. L. Day S. E. Piaggi P. Wiedrich K. (2024). Identification and functional validation of rare coding variants in genes linked to monogenic obesity. Obesity 32, 1769–1777. 10.1002/oby.24101 39192769 PMC11361714

[B69] Krude H. Biebermann H. Luck W. Horn R. Brabant G. Grüters A. (1998). Severe early-onset obesity, adrenal insufficiency and red hair pigmentation caused by POMC mutations in humans. Nat. Genet. 19, 155–157. 10.1038/509 9620771

[B70] Kumar R. Ningombam S. S. Kumar R. Goel H. Gogia A. Khurana S. (2022). Comprehensive mutations analyses of FTO (fat mass and obesity-associated gene) and their effects on FTO’s substrate binding implicated in obesity. Front. Nutr. 9, 852944. 10.3389/fnut.2022.852944 35923209 PMC9339907

[B71] Künzel R. Faust H. Bundalian L. Blüher M. Jasaszwili M. Kirstein A. (2025). Detecting monogenic obesity: a systematic exome-wide workup of over 500 individuals. Int. J. Obes. 49, 1400–1411. 10.1038/s41366-025-01819-0 40523925 PMC12283394

[B72] Lan N. Lu Y. Zhang Y. Pu S. Xi H. Nie X. (2020). FTO – a common genetic basis for obesity and cancer. Front. Genet. 11, 559138. 10.3389/fgene.2020.559138 33304380 PMC7701174

[B73] Levy M. E. Telis N. Schiabor Barrett K. M. Bolze A. Stoller D. Chapman C. N. (2024). Influence of BMI-Associated genetic variants and metabolic risk factors on weight loss with semaglutide: a longitudinal clinico-genomic cohort study. 10.1101/2024.10.31.24316494

[B74] Li L. Liu D.-W. Yan H.-Y. Wang Z.-Y. Zhao S.-H. Wang B. (2016). Obesity is an independent risk factor for non‐alcoholic fatty liver disease: evidence from a meta‐analysis of 21 cohort studies. Obes. Rev. 17, 510–519. 10.1111/obr.12407 27020692

[B75] Li M.-H. Chen I.-C. Yang H.-W. Yen H.-C. Huang Y.-C. Hsu C.-C. (2024). The characterization and comorbidities of heterozygous Bardet-Biedl syndrome carriers. Int. J. Med. Sci. 21, 784–794. 10.7150/ijms.92766 38617006 PMC11008491

[B76] Lin F. Yu B. Ling B. Lv G. Shang H. Zhao X. (2023). Weight loss efficiency and safety of tirzepatide: a Systematic review. PLoS ONE 18, e0285197. 10.1371/journal.pone.0285197 37141329 PMC10159347

[B77] Lister N. B. Baur L. A. Felix J. F. Hill A. J. Marcus C. Reinehr T. (2023). Child and adolescent obesity. Nat. Rev. Dis. Prim. 9, 24. 10.1038/s41572-023-00435-4 37202378

[B78] Löffler D. Behrendt S. Creemers J. W. M. Klammt J. Aust G. Stanik J. (2017). Functional and clinical relevance of novel and known PCSK1 variants for childhood obesity and glucose metabolism. Mol. Metab. 6, 295–305. 10.1016/j.molmet.2016.12.002 28271036 PMC5323889

[B79] Loos R. J. F. (2025). Genetic causes of obesity: mapping a path forward. Trends Mol. Med. 31, 319–325. 10.1016/j.molmed.2025.02.002 40089418

[B80] Loos R. J. F. Yeo G. S. H. (2022). The genetics of obesity: from discovery to biology. Nat. Rev. Genet. 23, 120–133. 10.1038/s41576-021-00414-z 34556834 PMC8459824

[B81] Ma X. Liu R. Pratt E. J. Benson C. T. Bhattachar S. N. Sloop K. W. (2024). Effect of food consumption on the pharmacokinetics, safety, and tolerability of once-daily orally administered Orforglipron (LY3502970), a non-peptide GLP-1 receptor agonist. Diabetes Ther. 15, 819–832. 10.1007/s13300-024-01554-1 38402332 PMC10951152

[B82] Mahmoud R. Kimonis V. Butler M. G. (2022). Genetics of obesity in humans: a clinical review. IJMS 23, 11005. 10.3390/ijms231911005 36232301 PMC9569701

[B83] Masip G. Han H. Y. Meng T. Nielsen D. E. (2025). Polygenic risk and nutrient intake interactions on obesity outcomes: a systematic review and meta‐analysis of observational studies. Obes. Rev. 26, e13941. 10.1111/obr.13941 40375759 PMC12318916

[B84] McEwan A. R. Hing B. Erickson J. C. Hutchings G. Urama C. Norton-Hughes E. (2024). An ancient polymorphic regulatory region within the BDNF gene associated with obesity modulates anxiety-like behaviour in mice and humans. Mol. Psychiatry 29, 660–670. 10.1038/s41380-023-02359-7 38228888 PMC11153140

[B85] Mendiratta M. S. Yang Y. Balazs A. E. Willis A. S. Eng C. M. Karaviti L. P. (2011). Early onset obesity and adrenal insufficiency associated with a homozygous POMC mutation. Int. J. Pediatr. Endocrinol. 5, 5. 10.1186/1687-9856-2011-5 21860632 PMC3159139

[B86] Menon R. Khan N. Charugulla S. Bassi A. Dangre P. Dedaniya A. (2024). Validation of a genome-wide polygenic score for obesity in South Asians. 10.21203/rs.3.rs-4460496/v1 PMC12441606 40969326

[B87] Merkestein M. Laber S. McMurray F. Andrew D. Sachse G. Sanderson J. (2015). FTO influences adipogenesis by regulating mitotic clonal expansion. Nat. Commun. 6, 6792. 10.1038/ncomms7792 25881961 PMC4410642

[B88] Miksza U. Adamska-Patruno E. Bauer W. Fiedorczuk J. Czajkowski P. Moroz M. (2023). Obesity-related parameters in carriers of some BDNF genetic variants may depend on daily dietary macronutrients intake. Sci. Rep. 13, 6585. 10.1038/s41598-023-33842-4 37085692 PMC10121660

[B89] Mohammed I. Haris B. Al-Barazenji T. Vasudeva D. Tomei S. Al Azwani I. (2023). Understanding the genetics of early-onset obesity in a cohort of children from Qatar. J. Clin. Endocrinol. and Metabolism 108, 3201–3213. 10.1210/clinem/dgad366 37329217 PMC10655519

[B90] Mohammed I. Selvaraj S. Ahmed W. S. Al-Barazenji T. Dauleh H. Love D. R. (2024). Functional evaluation of a novel homozygous ADCY3 variant causing childhood obesity. IJMS 25, 11815. 10.3390/ijms252111815 39519366 PMC11547096

[B91] Mohammed I. Ahmed W. S. Al-Barazenji T. Dauleh H. Love D. R. Hussain K. (2026). Novel SIM1 variants expanding the spectrum of SIM1-Related obesity. IJMS 27, 533. 10.3390/ijms27010533 41516406 PMC12786438

[B92] Mohn A. Di Ludovico A. Polidori N. Giannini C. Di Pietro G. Lauriola F. (2025). MC4-R variant confirms its association with obesity during progression from childhood to adolescence. Sci. Rep. 15, 13045. 10.1038/s41598-025-96408-6 40240490 PMC12003884

[B93] Naeem M. Imran L. Banatwala U. E. S. S. (2024). Unleashing the power of retatrutide: a possible triumph over obesity and overweight: a correspondence. Health Sci. Rep. 7, e1864. 10.1002/hsr2.1864 38323122 PMC10844714

[B94] Niazi R. K. Gjesing A. P. Hollensted M. Have C. T. Grarup N. Pedersen O. (2018). Identification of novel LEPR mutations in Pakistani families with morbid childhood obesity. BMC Med. Genet. 19, 199. 10.1186/s12881-018-0710-x 30442103 PMC6238292

[B95] Nunziata A. Funcke J.-B. Borck G. Von Schnurbein J. Brandt S. Lennerz B. (2019). Functional and phenotypic characteristics of human Leptin receptor mutations. J. Endocr. Soc. 3, 27–41. 10.1210/js.2018-00123 30560226 PMC6293235

[B96] Parra E. J. Mazurek A. Gignoux C. R. Sockell A. Agostino M. Morris A. P. (2017). Admixture mapping in two Mexican samples identifies significant associations of locus ancestry with triglyceride levels in the BUD13/ZNF259/APOA5 region and fine mapping points to rs964184 as the main driver of the association signal. PLoS ONE 12, e0172880. 10.1371/journal.pone.0172880 28245265 PMC5330487

[B97] Perrone L. Marzuillo P. Grandone A. Del Giudice E. M. (2010). Chromosome 16p11.2 deletions: another piece in the genetic puzzle of childhood obesity. Ital. J. Pediatr. 36, 43. 10.1186/1824-7288-36-43 20540750 PMC2903605

[B98] Pitman J. L. Wheeler M. C. Lloyd D. J. Walker J. R. Glynne R. J. Gekakis N. (2014). A gain-of-function mutation in adenylate cyclase 3 protects mice from diet-induced obesity. PLoS ONE 9, e110226. 10.1371/journal.pone.0110226 25329148 PMC4199629

[B99] Poosri S. Boonyuen U. Chupeerach C. Soonthornworasiri N. Kwanbunjan K. Prangthip P. (2024). Association of FTO variants rs9939609 and rs1421085 with elevated sugar and fat consumption in adult obesity. Sci. Rep. 14, 25618. 10.1038/s41598-024-77004-6 39463443 PMC11514288

[B100] Pratt E. Ma X. Liu R. Robins D. Haupt A. Coskun T. (2023). Orforglipron (LY3502970), a novel, oral non‐peptide glucagon‐like peptide‐1 receptor agonist: a phase 1a, blinded, placebo‐controlled, randomized, single‐ and multiple‐ascending‐dose study in healthy participants. Diabetes Obes. Metab. 25, 2634–2641. 10.1111/dom.15184 37344954

[B101] Ramachandrappa S. Raimondo A. Cali A. M. G. Keogh J. M. Henning E. Saeed S. (2013). Rare variants in single-minded 1 (SIM1) are associated with severe obesity. J. Clin. Invest. 123, 3042–3050. 10.1172/JCI68016 23778139 PMC3696558

[B102] Ramos-Molina B. Martin M. G. Lindberg I. (2016). “PCSK1 variants and human obesity,” in Progress in Molecular Biology and Translational Science (Elsevier), 47–74. 10.1016/bs.pmbts.2015.12.001 PMC6082390 27288825

[B103] Richards S. Aziz N. Bale S. Bick D. Das S. Gastier-Foster J. (2015). Standards and guidelines for the interpretation of sequence variants: a joint consensus recommendation of the American college of medical genetics and genomics and the association for molecular pathology. Genet. Med. 17, 405–424. 10.1038/gim.2015.30 25741868 PMC4544753

[B104] Roussel-Gervais A. Sgroi S. Cambet Y. Lemeille S. Seredenina T. Krause K.-H. (2023). Genetic knockout of NTRK2 by CRISPR/Cas9 decreases neurogenesis and favors glial progenitors during differentiation of neural progenitor stem cells. Front. Cell. Neurosci. 17, 1289966. 10.3389/fncel.2023.1289966 38161998 PMC10757602

[B105] Rui L. (2014). SH2B1 regulation of energy balance, body weight, and glucose metabolism. WJD 5, 511–526. 10.4239/wjd.v5.i4.511 25126397 PMC4127586

[B106] Ryan D. H. Lingvay I. Deanfield J. Kahn S. E. Barros E. Burguera B. (2024). Long-term weight loss effects of semaglutide in obesity without diabetes in the SELECT trial. Nat. Med. 30, 2049–2057. 10.1038/s41591-024-02996-7 38740993 PMC11271387

[B107] Saeed S. Khanam R. Janjua Q. M. Manzoor J. Ning L. Hanook S. (2023). High morbidity and mortality in children with untreated congenital deficiency of leptin or its receptor. Cell Rep. Med. 4, 101187. 10.1016/j.xcrm.2023.101187 37659411 PMC10518629

[B108] Saeed S. Bonnefond A. Froguel P. (2025). Obesity: exploring its connection to brain function through genetic and genomic perspectives. Mol. Psychiatry 30, 651–658. 10.1038/s41380-024-02737-9 39237720 PMC11746128

[B109] Sahibdeen V. Crowther N. J. Soodyall H. Hendry L. M. Munthali R. J. Hazelhurst S. (2018). Genetic variants in SEC16B are associated with body composition in black South Africans. Nutr and Diabetes 8, 43. 10.1038/s41387-018-0050-0 30026463 PMC6053407

[B110] Shi R. Lu W. Tian Y. Wang B. (2023). Intestinal SEC16B modulates obesity by regulating chylomicron metabolism. Mol. Metab. 70, 101693. 10.1016/j.molmet.2023.101693 36796587 PMC9976576

[B111] Singh G. M. Danaei G. Farzadfar F. Stevens G. A. Woodward M. Wormser D. (2013). Global burden of metabolic risk factors of chronic diseases collaborating group; Asia-Pacific cohort studies collaboration (APCSC), diabetes epidemiology: collaborative analysis of diagnostic criteria in Europe (DECODE), emerging risk factor collaboration (ERFC), prospective studies collaboration (PSC), 2013. The age-specific quantitative effects of metabolic risk factors on cardiovascular diseases and diabetes: a pooled analysis. PLoS ONE 8, e65174. 10.1371/journal.pone.0065174 23935815 PMC3728292

[B112] Smit R. A. J. Wade K. H. Hui Q. Arias J. D. Yin X. Christiansen M. R. (2025). Polygenic prediction of body mass index and obesity through the life course and across ancestries. Nat. Med. 31, 3151–3168. 10.1038/s41591-025-03827-z 40691366 PMC12443623

[B113] Sobreira D. R. Joslin A. C. Zhang Q. Williamson I. Hansen G. T. Farris K. M. (2021). Extensive pleiotropism and allelic heterogeneity mediate metabolic effects of *IRX3* and *IRX5* . Science 372, 1085–1091. 10.1126/science.abf1008 34083488 PMC8386003

[B114] Son J. E. Dou Z. Kim K.-H. Hui C.-C. (2022). Deficiency of Irx5 protects mice from obesity and associated metabolic abnormalities. Int. J. Obes. 46, 2029–2039. 10.1038/s41366-022-01221-0 36115924

[B115] Sridhar G. R. Gumpeny L. (2024). Melanocortin 4 receptor mutation in obesity. World J. Exp. Med. 14, 99239. 10.5493/wjem.v14.i4.99239 39713072 PMC11551707

[B116] Stanikova D. Buzga M. Krumpolec P. Skopkova M. Surova M. Ukropcova B. (2017). Genetic analysis of single-minded 1 gene in early-onset severely obese children and adolescents. PLoS ONE 12, e0177222. 10.1371/journal.pone.0177222 28472148 PMC5417716

[B117] Stijnen P. Ramos-Molina B. O’Rahilly S. Creemers J. W. M. (2016). PCSK1 mutations and human endocrinopathies: from obesity to gastrointestinal disorders. Endocr. Rev. 37, 347–371. 10.1210/er.2015-1117 27187081

[B118] Strobel A. Issad T. Camoin L. Ozata M. Strosberg A. D. (1998). A leptin missense mutation associated with hypogonadism and morbid obesity. Nat. Genet. 18, 213–215. 10.1038/ng0398-213 9500540

[B119] Supti D. A. Akter F. Rahman M. I. Munim M. A. Tonmoy M. I. Q. Tarin R. J. (2024). Meta-analysis investigating the impact of the LEPR rs1137101 (A>G) polymorphism on obesity risk in Asian and Caucasian ethnicities. Heliyon 10, e27213. 10.1016/j.heliyon.2024.e27213 38496879 PMC10944198

[B120] Tas A. Atabey M. Gokcen P. Ozel M. İ. Karagoz Z. K. Ugur K. (2022). Leptin/melanocortin pathway hormones in obese patients after laparoscopic sleeve gastrectomy. Eur. Rev. Med. Pharmacol. Sci. 26, 1484–1491. 10.26355/eurrev_202203_28212 35302192

[B121] Temaj G. Nuhii N. Sayer J. A. (2022). The impact of consanguinity on human health and disease with an emphasis on rare diseases. J. Rare Dis. 1, 2. 10.1007/s44162-022-00004-5

[B122] Thompson D. Edelsberg J. Colditz G. A. Bird A. P. Oster G. (1999). Lifetime health and economic consequences of obesity. Arch. Intern Med. 159, 2177–2183. 10.1001/archinte.159.18.2177 10527295

[B123] Tirthani E. Said M. S. Rehman A. (2025). “Genetics and obesity,” in StatPearls (Treasure Island (FL): StatPearls Publishing).

[B124] Toumba M. Fanis P. Vlachakis D. Neocleous V. Phylactou L. Skordis N. (2021). Molecular modelling of novel ADCY3 variant predicts a molecular target for tackling obesity. Int. J. Mol. Med. 49, 10. 10.3892/ijmm.2021.5065 34821371 PMC8651229

[B125] Trang K. Grant S. F. A. (2023). Genetics and epigenetics in the obesity phenotyping scenario. Rev. Endocr. Metab. Disord. 24, 775–793. 10.1007/s11154-023-09804-6 37032403 PMC10088729

[B126] Tzoulis P. Batavanis M. Baldeweg S. (2024). A real-world study of the effectiveness and safety of semaglutide for weight loss. Cureus 16, e59558. 10.7759/cureus.59558 38826889 PMC11144277

[B127] Urabe H. Kojima H. Chan L. Terashima T. Ogawa N. Katagi M. (2013). Haematopoietic cells produce BDNF and regulate appetite upon migration to the hypothalamus. Nat. Commun. 4, 1526. 10.1038/ncomms2536 23443554 PMC3640364

[B128] Van Uhm J. Van Rossum E. F. C. Van Haelst M. M. Jansen P. R. Van Den Akker E. L. T. (2025). Polygenic childhood obesity: integrating genetics and environment for early intervention. Horm. Res. Paediatr. 99 (2), 228–236. 10.1159/000546951 40527292 PMC12266691

[B129] Vázquez-Moreno M. Locia-Morales D. Valladares-Salgado A. Sharma T. Perez-Herrera A. Gonzalez-Dzib R. (2021). The MC4R p.Ile269Asn mutation confers a high risk for type 2 diabetes in the Mexican population via obesity dependent and independent effects. Sci. Rep. 11, 3097. 10.1038/s41598-021-82728-w 33542413 PMC7862248

[B130] Verde L. Galasso M. Coletta D. K. Savastano S. Mandarino L. J. Colao A. (2025). The interplay of UCP3 and PCSK1 variants in severe obesity. Curr. Obes. Rep. 14, 38. 10.1007/s13679-025-00631-1 40281302 PMC12031958

[B131] Wadden T. A. Chao A. M. Machineni S. Kushner R. Ard J. Srivastava G. (2023). Tirzepatide after intensive lifestyle intervention in adults with overweight or obesity: the SURMOUNT-3 phase 3 trial. Nat. Med. 29, 2909–2918. 10.1038/s41591-023-02597-w 37840095 PMC10667099

[B132] Wade K. H. Lam B. Y. H. Melvin A. Pan W. Corbin L. J. Hughes D. A. (2021). Loss-of-function mutations in the melanocortin 4 receptor in a UK birth cohort. Nat. Med. 27, 1088–1096. 10.1038/s41591-021-01349-y 34045736 PMC7611835

[B133] Wang Y. Wang Q. J. (2004). The prevalence of prehypertension and hypertension among US adults according to the New Joint National Committee guidelines: New challenges of the old problem. Arch. Intern Med. 164, 2126–2134. 10.1001/archinte.164.19.2126 15505126

[B134] Wang Y. Pan L. Wan S. Yihuo W. Yang F. He H. (2022). MC4R gene polymorphisms interact with the urbanized living environment on obesity: results from the Yi migrant study. Front. Genet. 13, 849138. 10.3389/fgene.2022.849138 35495128 PMC9046839

[B135] Weiss T. Yang L. Carr R. D. Pal S. Sawhney B. Boggs R. (2022). Real-world weight change, adherence, and discontinuation among patients with type 2 diabetes initiating glucagon-like peptide-1 receptor agonists in the UK. BMJ Open Diab Res. Care 10, e002517. 10.1136/bmjdrc-2021-002517 35101924 PMC8804648

[B136] Wong H.S.-C. Tsai S.-Y. Chu H.-W. Lin M.-R. Lin G.-H. Tai Y.-T. (2022). Genome-wide association study identifies genetic risk loci for adiposity in a Taiwanese population. PLoS Genet. 18, e1009952. 10.1371/journal.pgen.1009952 35051171 PMC8853642

[B137] Wong H. J. Sim B. Teo Y. H. Teo Y. N. Chan M. Y. Yeo L. L. L. (2025). Efficacy of GLP-1 receptor agonists on weight loss, BMI, and waist circumference for patients with obesity or overweight: a systematic review, meta-analysis, and meta-regression of 47 randomized controlled trials. Diabetes Care 48, 292–300. 10.2337/dc24-1678 39841962

[B138] Wu L. Ma F. Zhao X. Zhang M.-X. Wu J. Mi J. (2019). *GNPDA2* gene affects adipogenesis and alters the transcriptome profile of human adipose-derived mesenchymal stem cells. Int. J. Endocrinol. 2019, 1–10. 10.1155/2019/9145452 31467530 PMC6701328

[B139] Yang Q. Xiao T. Guo J. Su Z. (2017). Complex relationship between obesity and the fat mass and obesity locus. Int. J. Biol. Sci. 13, 615–629. 10.7150/ijbs.17051 28539834 PMC5441178

[B140] Yanovski J. Angel M.-M. G. Malhotra S. Yuan G. Chung W. Dollfus H. (2024). 3-year setmelanotide weight outcomes in patients with bardet-biedl syndrome and obesity. Endocrine Abst. 99, EP23. 10.1530/endoabs.99.EP23

[B141] Yeo G. S. H. Chao D. H. M. Siegert A.-M. Koerperich Z. M. Ericson M. D. Simonds S. E. (2021). The melanocortin pathway and energy homeostasis: from discovery to obesity therapy. Mol. Metab. 48, 101206. 10.1016/j.molmet.2021.101206 33684608 PMC8050006

[B142] Yonekawa S. Furuno A. Baba T. Fujiki Y. Ogasawara Y. Yamamoto A. (2011). Sec16B is involved in the endoplasmic reticulum export of the peroxisomal membrane biogenesis factor peroxin 16 (Pex16) in mammalian cells. Proc. Natl. Acad. Sci. U.S.A. 108, 12746–12751. 10.1073/pnas.1103283108 21768384 PMC3150892

[B143] Yupanqui-Lozno H. Bastarrachea R. A. Yupanqui-Velazco M. E. Alvarez-Jaramillo M. Medina-Méndez E. Giraldo-Peña A. P. (2019). Congenital leptin deficiency and leptin gene missense mutation found in two Colombian sisters with severe obesity. Genes 10, 342. 10.3390/genes10050342 31067764 PMC6562380

[B144] Zhao Y. Hong N. Liu X. Wu B. Tang S. Yang J. (2014). A novel mutation in *Leptin* Gene is associated with severe obesity in Chinese individuals. BioMed Res. Int. 2014, 1–3. 10.1155/2014/912052 24707501 PMC3953508

[B145] Zhong B. Nie N. Dong M. (2025). Molecular mechanisms of the obesity associated with bardet‐biedl syndrome: an update. Obes. Rev. 26, e13859. 10.1111/obr.13859 39477210

